# A retrospective case review of decedents recovered from aquatic environments in Cape Town, South Africa

**DOI:** 10.1007/s12024-026-01229-2

**Published:** 2026-05-28

**Authors:** Laura Jane Heathfield, Lisa Malan, Chandra Finaughty, Calvin Gerald Mole, Victoria Elaine Gibbon

**Affiliations:** 1 Division of Forensic Medicine and Toxicology, Department of Pathology, Faculty of Health Science, University of Cape Town, Anzio Road, Observatory, Cape Town, 7925 South Africa; 2 Division of Clinical Anatomy and Biological Anthropology, Department of Human Biology, University of Cape Town, Cape Town, South Africa

**Keywords:** Mortality data, Forensic human identification, Post-mortem submersion interval, Salt River Mortuary, Medico-legal death investigation

## Abstract

The identification of deceased individuals is a mandated component of medico-legal death investigations. Due to the unique taphonomic circumstances of decedents retrieved from aquatic environments, identification is often challenging. This study retrospectively examined 289 cases of deceased individuals recovered from aquatic environments that were admitted to Salt River Mortuary in Cape Town between January 2017 and December 2021. Case report data and associated documented information were analysed to evaluate reported post-mortem submersion intervals (PMSI), recovered body segments, and identification methods to inform future procedural improvements and prevention strategies. Findings indicated that 48.4% (140/289) of cases were recovered from the ocean. Overall, 33.2% (96/289) of cases were admitted to the mortuary with unknown identity, among these, 25.0% (24/96) were not identified after the medico legal investigation. The presumed identities of a further two cases were unconfirmed following post-mortem investigation, resulting in 9.0% (26/289) decedents remaining unidentified. Most unidentified decedents were recovered from the ocean 69.2% (18/26). Samples for DNA profiling were collected in 28.4% (82/289) cases and were successfully used in identification for 32.9% (27/82) cases. The average post-mortem submersion interval was less than two weeks for 51.2% (148/289) of cases but was undetermined for 47.8% (138/289) cases. The taphonomic environment of aquatic cases results in rapid and extensive breakdown of biological tissues leading to reduced rates of identification, which requires scientific focus and attention. To enhance identification rates for aquatic cases, further research into methodological and technological adjustments are required. In particular exploring alternatives to visual identification through use of DNA analyses and a better understanding of taphonomic processes in these aquatic environments may improve medico-legal death investigations.

**Supplementary Information:**

The online version contains supplementary material available at https://doi.org/10.1007/s12024-026-01229-2.

## Introduction

Identifying decedents from aquatic environments whether fresh, skeletonised, complete, or fragmented is important for familial closure and supporting legal processes [1, 2]. Factors such as political instability, family unawareness, service limitations, and circumstances of recovery—especially after submersion in an aquatic environment—can hinder identification [3].

Drowning is the seventh leading cause of unintentional injury death globally and the top cause of death in children under four [4]. While most aquatic deaths result from drowning, other causes such as hypothermia, medical conditions, and trauma also contribute [4]. The United Nations Global Drowning Intervention Plan highlights that over 90% of drowning deaths occur in low- and middle-income countries, with Africa having the highest rates and Asia the greatest number of deaths [5]. In fact, Africa had one of the highest drowning rates in 2021 despite under-reporting [6]. Natural disasters and migration also add to the number of decedents recovered from aquatic systems [7–9], whereby recovery and identification are hampered by severe oceanic conditions [10].

In South Africa, most accidental drownings in the ocean occur during the summer season when coastal visitation increases and more people are on leave, with approximately only 15% of the population able to swim [11, 12]. The coastline also sees homicide victims wash ashore, as the ocean can conceal murders [13–19]. Risks are heightened in non-metro areas with limited safety measures, especially near informal settlements. While large-scale water migration into South Africa is rare, there were 680 migrant-related drowning deaths reported between 2014 and 2021—mostly due to river crossings from neighbouring countries [16, 20].

Aquatic environments complicate identification due to environmental exposure and accelerated decomposition, often causing severe tissue degradation [21]. Conditions such as scavenging, dismemberment, and skin slippage can make fingerprinting impossible [22–24]. In these cases, DNA analysis is often required to confirm identification, and in some instances, to match dismembered body segments [25, 26]. Despite advancing DNA technologies, post-mortem aquatic conditions can still limit the success of forensic DNA profiling [27].

Improving identification processes for decedents from aquatic environments requires an understanding of case circumstances, recovery settings, and identification outcomes. Analysing past cases reveals patterns that aid protocol development and case resolution. This Africa-based retrospective review of cases from Salt River Mortuary (now replaced with the Observatory Forensic Pathology Institute) in Cape Town—one of South Africa’s busiest—offers baseline insights into the taphonomic complexities affecting identification in medico-legal investigations for aquatic-related deaths. We aimed to use retrospective case report data to examine the reported post-mortem submersion intervals (PMSI), recovered body segments, and what identification methods were used as well as their associated success in relation to documented recovery circumstances (PMSI, taphonomy, case context). The results of which would guide future procedural improvements and prevention strategies in aquatic death investigations.

## Methodology

### Study design and approvals

This retrospective, descriptive, cross-sectional analysis employed a quantitative research paradigm. Case reports from the Salt River Mortuary in Cape Town, South Africa between 1 January 2017 and 31 December 2021 involving decedents recovered from aquatic environments, or those suspected to have been submerged post-mortem, were examined. No cases were directly investigated by the authors and the only data available for review were that in the written and completed forensic pathologist case reports. Ethical approval was obtained from the University of Cape Town Faculty of Health Sciences Human Research Ethics Committee (HREC: 434/2022).

### Environmental description

All cases were retrieved from Cape Town, South Africa and its surrounding area. Cape Town’s coastline is shaped by the meeting of two major ocean currents: the cold Benguela Current from the Atlantic Ocean and the warm Agulhas Current from the Indian Ocean. Along the west coast (e.g. Blouberg and the Atlantic Seaboard), the Benguela Current brings cold, nutrient-rich waters, with temperatures often sitting between 10 and 15 °C, especially in summer due to upwelling. In contrast, the False Bay area on the eastern side of the Cape Peninsula is influenced slightly by the Agulhas Current, which can experience milder waters, typically around 16–22 °C in summer. These environmental differences are important when considering drowning cases and the effect of fluctuating sea temperatures on taphonomic processes.

Further to the seawater environment, of note is that most of the population of Cape Town live within 30 min of travel to the coastline, with a high proportion of the population being from in low-income households, and residing in informal settlements. This is of relevance when considering the drowning risk to the general population due to proximity to the ocean, and in low resource areas, the potential of insufficient water safety training and awareness.

### Case identification and data collection

The Salt River Mortuary’s post-mortem report database was searched and assessed for relevant cases that met the study’s inclusion criteria (Table 1). All data collected were directly reported from the case files and no new investigations were undertaken related to these cases by the authors. As related to the inclusion criteria, all cases wherein decedents were recovered from aquatic environments, or suspected to have been immersed in water, were included. Case reports where there was no recovery location documented, and those involving non-viable foetuses were excluded. Cases that met the study’s inclusion criteria (*n* = 289) were then analysed further for demographic information, recovery locations, post-mortem findings (e.g., post-mortem submersion interval, body condition, cause of death), and identification attempts (Supplementary Table A). It is crucial to note that manners of deaths were based on circumstantial information and were determined by the forensic pathologist. Manner of death was not confirmed by a court of law outcome or concluded by the authors. For unidentified decedents, some case reports only provided age estimates, while others noted the presence of identification documents, which was then used to determine the age of identified individuals.

**Table 1:** Identification of cases via filters and keyword searches on the Salt River Mortuary Autopsy Database

Case identification	Type of death = drowning (*n* = 186) Scene of death = river; sea; ocean; beach; lake; formal house outside; swimming pool; canal; dam; drain; ditch; harbour; informal toilets; sea/beach; stream; water reservoir (*n* = 163) Key words = river; water; bath; beach; canal; drown; submerge/submersion; toilet; dump; shower; stream; pool; immerse/immersion; sink/zink; marshland (*n* = 45)
Screening	Cases sought for retrieval (***n*** = 394) ⇒ Cases not retrieved (***n*** = 35) Cases assessed for eligibility (***n*** = 359) ⇒ Cases excluded (***n*** = 71)
Included	Cases included in review (***n*** = 289)

The PMSI reported in case documents were detailed to be either determined from the ‘last seen alive dates’, or in cases where this information was missing, it was noted that estimates were provided through forensic anthropological examination. For the retrospective case review, the documented aquatic environments were categorised into three broad groups for statistical analyses: ‘Ocean’, ‘Natural freshwater’ (dams, canals, and rivers), and ‘Urban water sources’ (sewerages and swimming pools). It is acknowledged that the biological and chemical conditions of the urban water sources are inconsistent, however, for the purpose of the retrospective case review, these were combined to distinguish them from natural fresh water and oceanic environments. Furthermore, without prospective water sampling and analysis of fresh-water sites, exact compositional differences could not be ascertained. This was also outside the scope of the study.

### Data and statistical analyses

All data were captured in a Microsoft™ Excel spreadsheet with active data validation and imported into R-studio for analysis, while SPSS© was utilised for data visualisation. Geo-coordinate heatmaps were generated using a free online tool (www.maptive.com; date accessed: 03/06/2024) to display the range of geographical locations. Chi-square tests were employed to assess differences in categorical data, with Fisher’s exact used when 2 × 2 Chi-square assumptions were violated. Where omnibus tests were significant, pairwise post-hoc analysis with Bonferroni correction was applied to assess difference in pairwise proportions. Effect size was estimated using Cramer’s V. No significant differences were observed in the proportion of cases with missing data (of each assessed categorical variable) for each water source assessed. As such the data was assumed to be missing completely at random, and cases with missing data for specific variables were excluded from analysis on that variable.

## Results

### Circumstances surrounding death

**Fig. 1:**
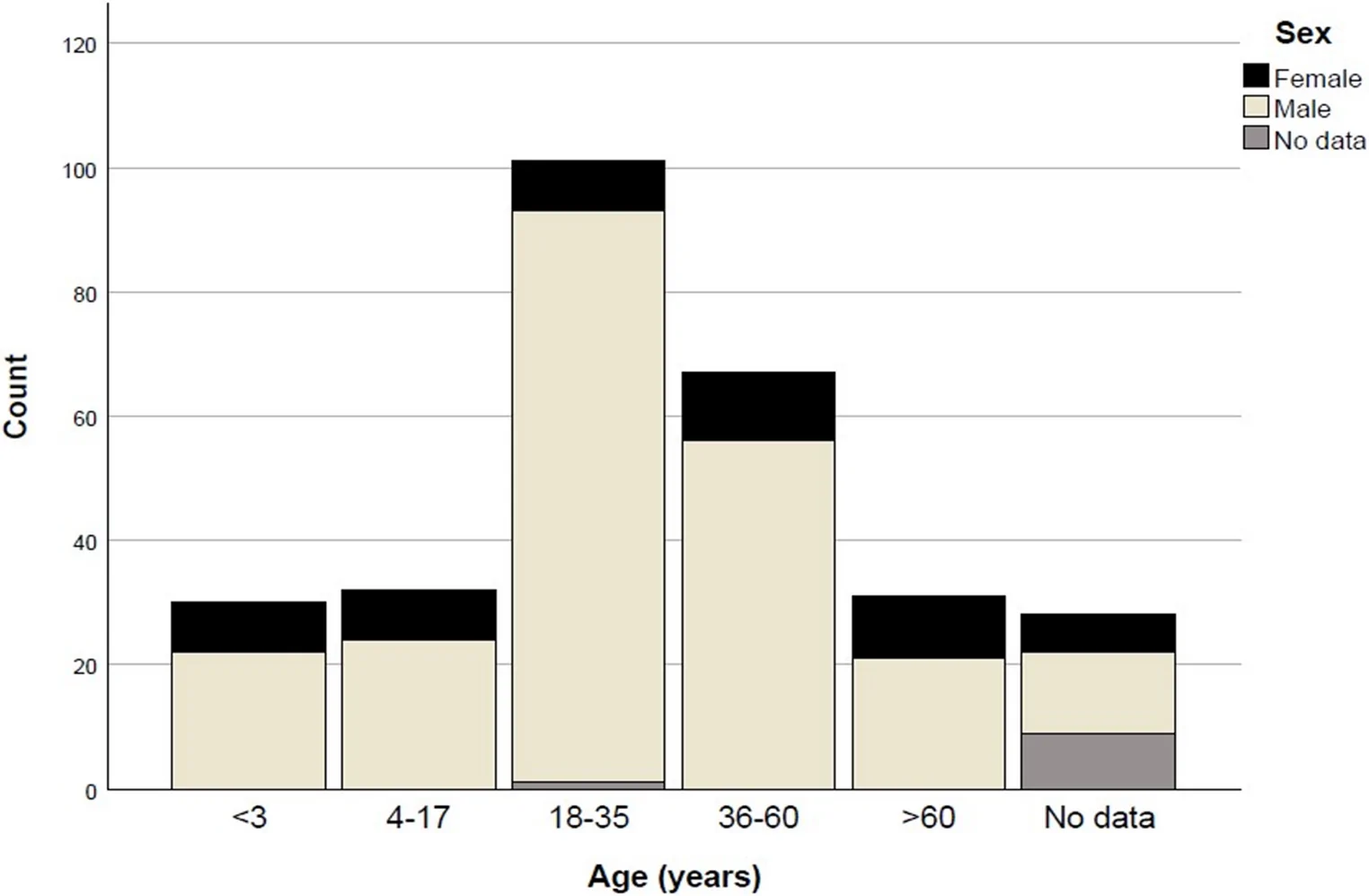
Distribution of water-logged decedents admitted to Salt River Mortuary from 2017–2021 according to age and sex. “No data” on x-axis – age not estimated for these cases. “No data” in key box – sex was not determined for these cases

Out of 19 369 cases admitted to Salt River Mortuary from 2017 to 2021, 289 case reports met the inclusion criteria (Table 1). Annual cases for decedents retrieved from aquatic environments ranged from 46 to 70 and averaged at 58 cases per year. Of these, 78.9% (228/289) were documented as male, 17.6% (51/289) female, and 3.5% (10/289) remained undetermined, and were reported as such (Fig. 1). Most individuals were reported to be over 18 years of age (199/289; 68.8%), with 50.8% (101/199) aged between 18 and 35 years (Fig. 1). Approximately 10.4% (30/289) were noted as under three years-of-age, and these cases were primarily found in urban water sources environments, such as swimming pools.

Approximately 55.4% (160/289) of cases were attributed to drowning, while 25.6% (74/289) remained undetermined, and 4.2% (12/289) were still under investigation, as documented in the case reports (Table 2). Traumatic causes accounted for 12.1% (35/289) of deaths, natural causes for 1.73% (5/289), asphyxia for 1.0% (3/289), and hypothermia for 0.7% (2/289), all of which were ascertained through post-mortem examinations conducted by forensic pathologists. Alleged manner of death as detailed in the reports, and determined by the forensic pathologist, was noted as primarily accidental (51.9%; 150/289), followed by undetermined (27.3%; 79/289), homicide (10.4%; 30/289), natural (6.6%; 19/289), and suicide (3.8%; 11/289) (Table 2).

**Table 2:** The number of cases and the percentage of cases for each category in different groups of information collected

Description	Number of cases	Percentage of cases
Water Category		
Ocean	140	48.4%
Natural freshwater	75	26.0%
Urban water sources	74	25.6%
**Total**	**289**	**100%**
**Cause of Death**		
Asphyxia	3	1.0%
Traumatic injuries	35	12.1%
Drowning	160	55.4%
Natural	5	1.7%
Under investigation	12	4.2%
Undetermined	74	25.6%
**Total**	**289**	**100%**
**Alleged Manner of Death**		
Accidental	150	51.9%
Natural	19	6.6%
Homicide	30	10.4%
Suicide	11	3.8%
Undetermined	79	27.3%
**Total**	**289**	**100%**
**Post-mortem Interval**		
< 24 h	118	40.8%
2–7 days	25	8.7%
8–14 days	5	1.7%
> 15 days	3	1.0%
Undetermined	138	47.4%
**Total**	**289**	**100%**
**Body Completeness**		
Full	248	85.8%
Partial	19	6.6%
Unspecified	22	7.6%
**Total**	**289**	**100%**
**State of Decomposition**		
Fresh	201	69.6%
Decomposed	53	18.3%
Skeletonised	11	3.8%
Unspecified	24	8.3%
**Total**	**289**	**100%**
**Presumed Identification**		
Yes	193	66.8%
No	96	33.2%
**Total**	**289**	**100%**
**Final Identification**		
Identified	263	91.0%
Unidentified	26	9.0%
**Total**	**289**	**100%**

### Body recovery

**Fig. 2:**
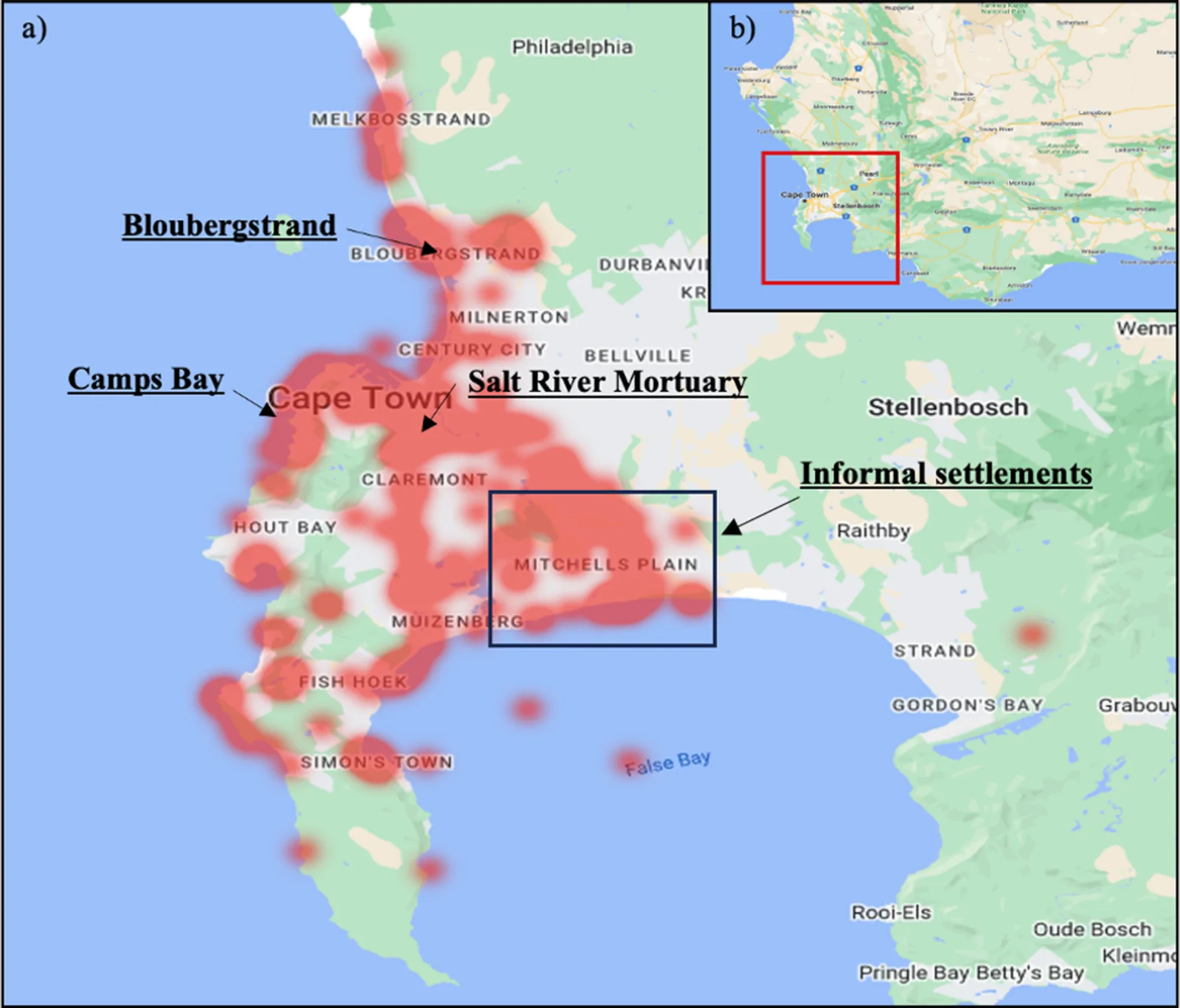
Water categories where decedents were recovered, along with water types that make up natural freshwater and urban water sources categories

**Fig. 3:**
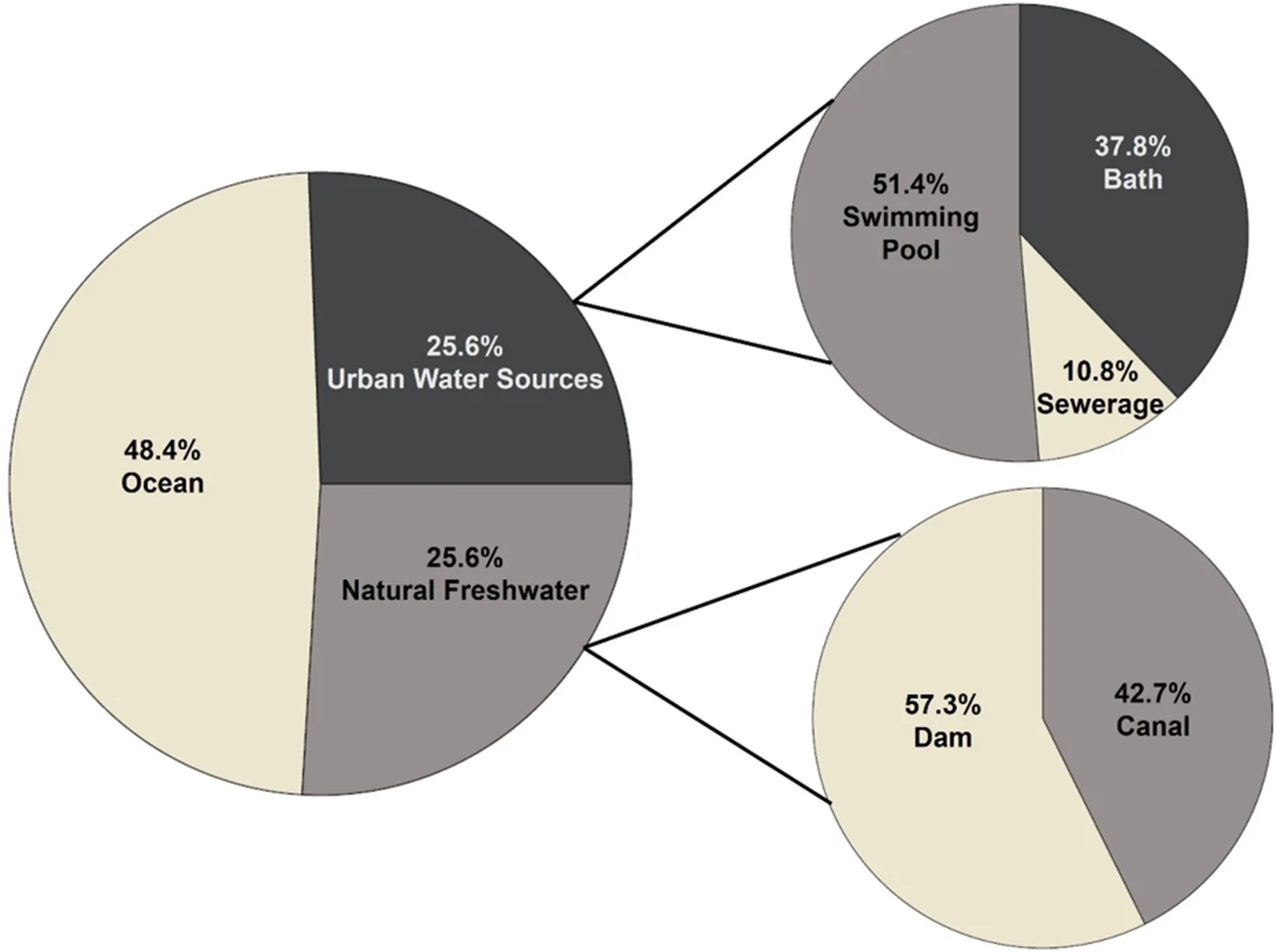
**a**. Heat map indicating locations where the cases analysed in this study were recovered by Forensic Pathology Services. The size of the circles indicates the relative number of cases originating from that area, with most cases being recovered along the coastline. **b**. A map of South Africa showing the focus area of this study in orange. Google Maps was used as a base map (www.google.com/maps)

The location of decedent recovery were documented in the case reports as co-ordinates. These co-ordinates were plotted using a heat map to evaluate drowning hotspots (Fig. 2a). Of the included cases, 48.8% (141/289) were recovered along the coastline, primarily along the western coast from Camps Bay to Bloubergstrand (48.6%, 69/141) (Figs. 2a and 3).

Additionally, 26.0% (75/289) were recovered from natural freshwater and 25.6% (74/289) from urban water sources, mainly from highly populated informal settlements (Fig. 2a). Decedents recovered from natural freshwater, it was noted that 57.3% (43/75) were from dams and 42.7% (32/75) were retrieved from canals and rivers. For urban water sources, 51.4% (38/74) were documented to be recovered from swimming pools, 37.8% (28/74) from baths, and 10.8% (8/74) from sewerage (Fig. 3).

An estimated PMSI was available and documented in 52.2% (151/289) of case reports, with no significant difference in the proportion of cases with an estimated PMSI recovered from the different water sources [X^2^(df = 2, *N* = 289) = 0.896, *p* = 0.639,* Cramer’s V = 0.056*]. In most cases the estimated PMSI was reported to be less than 24 h (78.1%; 118/151) (Table 3) as determined through information derived from the death investigation at the time. In cases where an estimated PMSI was available, there was a significant association between the estimated PMSI of individuals recovered from different water sources [Fisher’s Exact test, *p* = 0.007,* Cramer’s V = 0.242*]. While oceanic cases tended to have greater proportions of cases with longer PMSI compared to natural fresh and urban water sources, following pairwise comparison with Bonferroni correction, the only significant difference observed was a lower proportion of oceanic cases with a PMSI less than 24 h compared to freshwater cases [*Z* = 3.47, *p* = 0.006 (Bonferroni adjusted)]. PMSI was significantly associated with body completeness [Fisher’s Exact test, *p* < 0.001,* Cramer’s V = 0.891*] and state of decomposition [Fisher’s Exact test, *p* < 0.001,* Cramer’s V = 0.714]*, as partial, decomposed, and skeletonised individuals were more prevalent in cases with longer PMSIs (Table 2). The remaining 138 cases had PMSI of an undetermined amount of time.

**Table 3:** Number of cases according to alleged and final identification status per post-mortem submersion interval category

Alleged identity		Post-mortem submersion interval						
	Final identity	< 24 h	2–7 days	8–14 days	> 15 days	No data	Total	
Yes	Identified	105	16	2	0	68	191	66.1%
	Unidentified	0	2	0	0	0	2	0.7%
	*Sub-total*	105	18	2	0	68	193	
No	Identified	13	6	2	2	49	72	24.9%
	Unidentified	0	1	1	1	21	24	8.3%
	*Sub-total*	13	7	3	3	70	96	
	**Total**	**118**	**25**	**5**	**3**	**138**	**289**	
		**40.8%**	**8.7%**	**1.7%**	**1.0%**	**47.8%**		**100%**

### Identification

Of the cases included 33.2% (96/289) were admitted to Salt River Mortuary without a probable identity, as reported. Among these, 25.0% (24/96) remained unidentified after the medico-legal death investigation (Table 2). Only two of the cases admitted with an alleged identity remained unidentified. Therefore, a total of 26 decedents remained unidentified after the formal investigation process. Where an alleged PMSI was available, a significant association was observed between PMSI and decedents admitted without an alleged identity [Fisher’s Exact test, *p* < 0.001,* Cramer’s V = 0.416*]. Post-hoc pairwise comparisons indicated significantly greater proportions of individuals admitted with no alleged identification and PMSI’s of 8–14 days [*Z* = 2.58, *p* = 0.04 (Bonferroni adjusted)], and greater than 15 days [*Z* = 3.84, *p* < 0.001 (Bonferroni adjusted)] and a significantly greater proportion of individuals admitted with an alleged ID and a PMSI less than 24 h [*Z* = 3.82, *p* < 0.001 (Bonferroni adjusted)]. A similar association was observed between PMSI and final identification following investigation [Fisher’s Exact test, *p* < 0.001,* Cramer’s V = 0.388*]. However post-hoc pairwise comparisons indicated a significantly greater proportion of individuals with no final identification where the PMSI was 2–7 days [*Z* = 2.66, *p* = 0.032 (Bonferroni adjusted)], a significantly greater proportion of individuals with no final identification where the PMSI was greater than 15 days [*Z* = 2.94, *p* = 0.0132 (Bonferroni adjusted)], and a significantly greater proportion of individuals with final identification where the PMSI was less than 24 h [*Z* = 4.30, *p* < 0.001 (Bonferroni adjusted)].

**Fig. 4:**
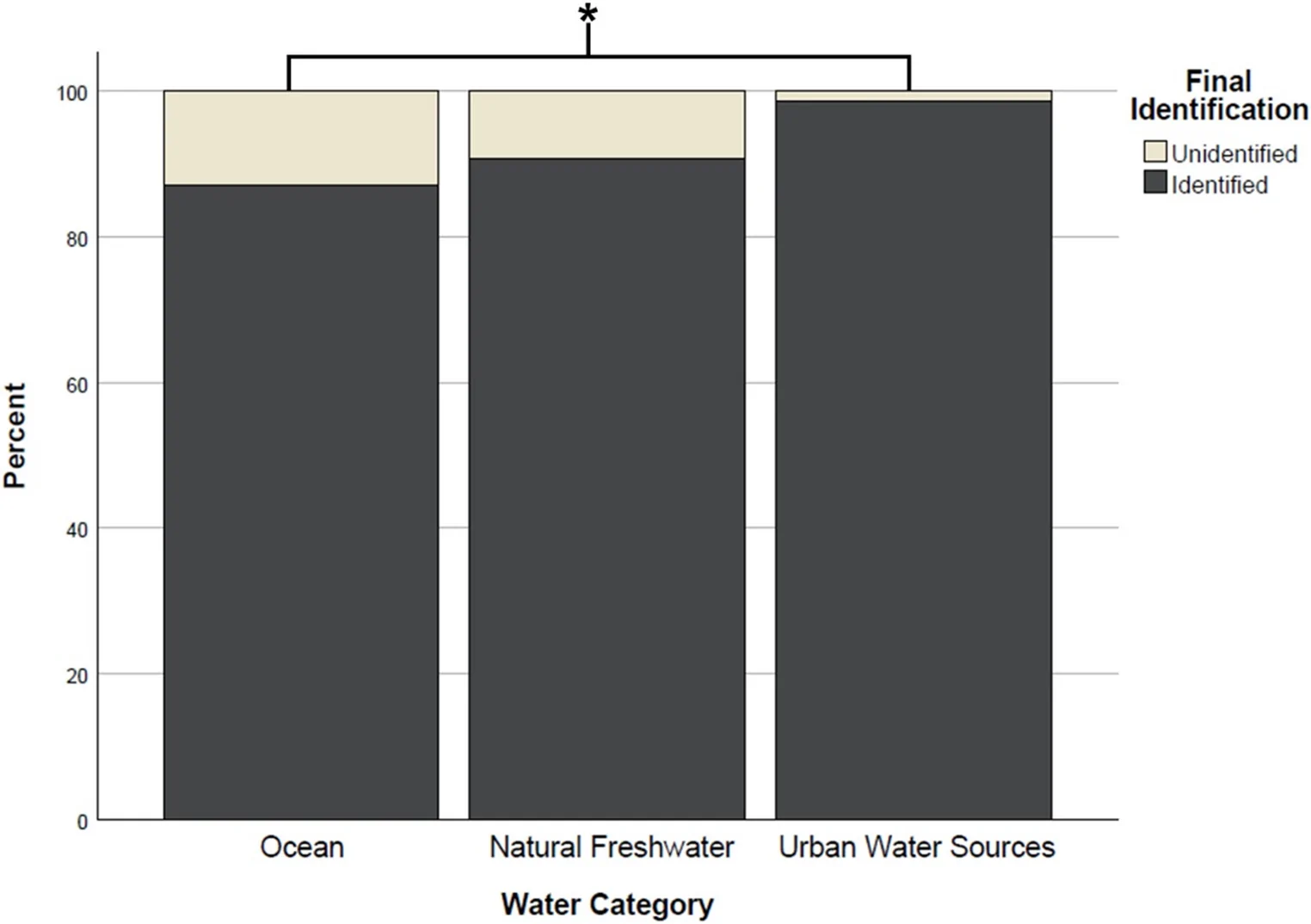
Bar chart showing identification success per water category. *Significant difference (*p* < 0.05; Bonferroni corrected) between proportion of unidentified recovered from ocean sources compared to urban water sources

Most unidentified decedents (69.2%; 18/26) were recovered from the ocean (Fig. 4). Of the remaining, 30.8% (8/26), seven were recovered from natural freshwater and only one from urban water sources. A significant association was observed between alleged identification and water category [X^2^(df = 2, *N* = 289) = 35.568, *p* < 0.001,* Cramer’s V = 0.351*]. Post-hoc pairwise comparisons indicated significantly greater proportions of individuals recovered from the ocean with no alleged identification compared to individuals recovered from urban water sources [*Z* = 5.94, *p* < 0.001 (Bonferroni adjusted)], and a significantly greater proportion of individuals recovered from the natural fresh water sources with no alleged identification compared to individuals recovered from urban water sources [*Z* = 4.89, *p* < 0.001 (Bonferroni adjusted)]. No significant difference was observed in the proportion of individuals recovered from the ocean with no alleged identification compared to individuals recovered from natural fresh water sources. Similarly, significant associations were observed between final identification and water source [X^2^(df = 2, *N* = 289) = 7.841, *p* = 0.02,* Cramer’s V = 0.165*]. Post-hoc pairwise comparisons indicated significantly greater proportions of individuals recovered from the ocean with no final identification compared to individuals recovered from urban water sources [*Z* = 2.81, *p* = 0.0147 (Bonferroni adjusted)].

**Fig. 5:**
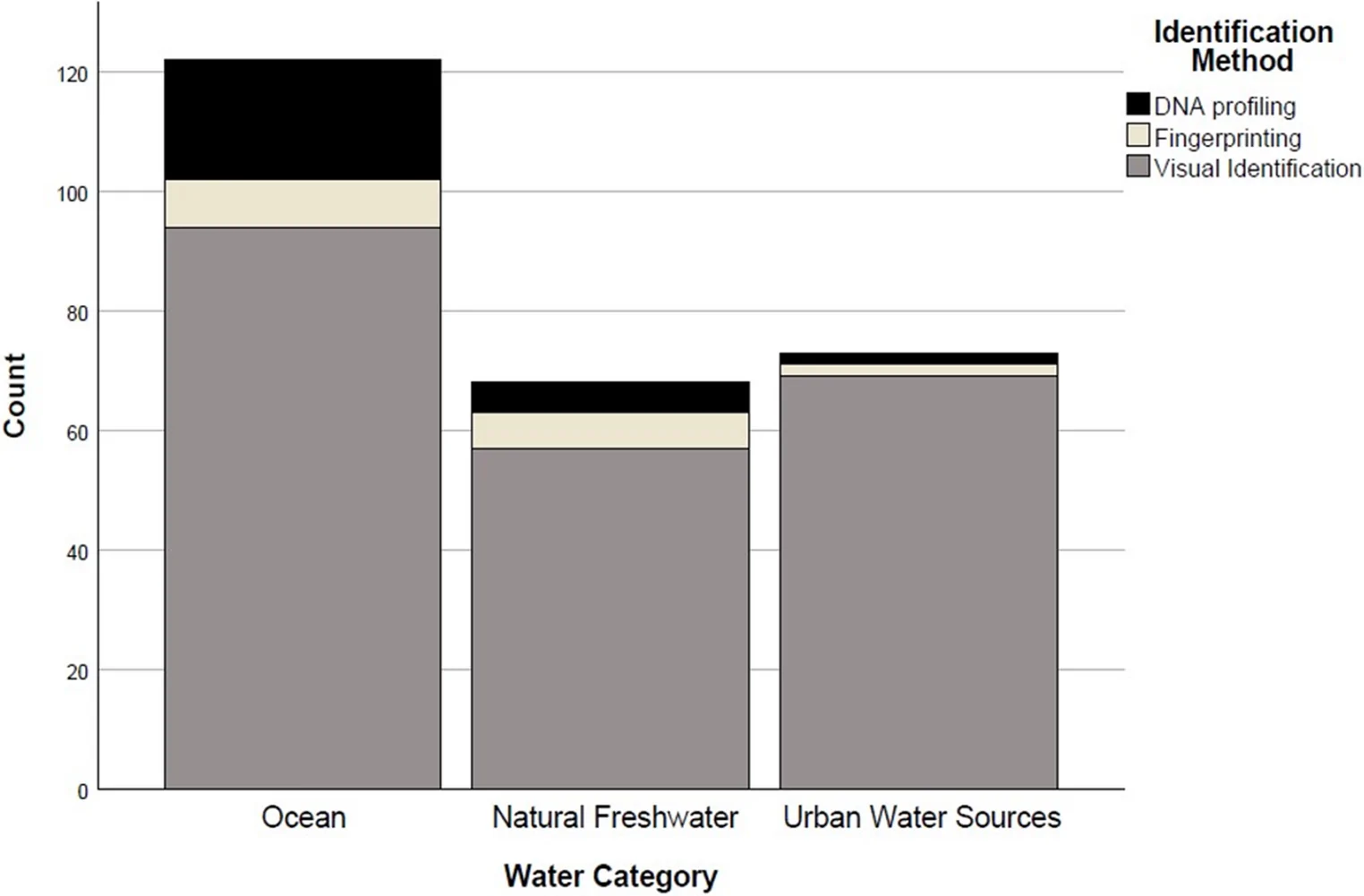
Method used to successfully identify decedents per water category

**Fig. 6:**
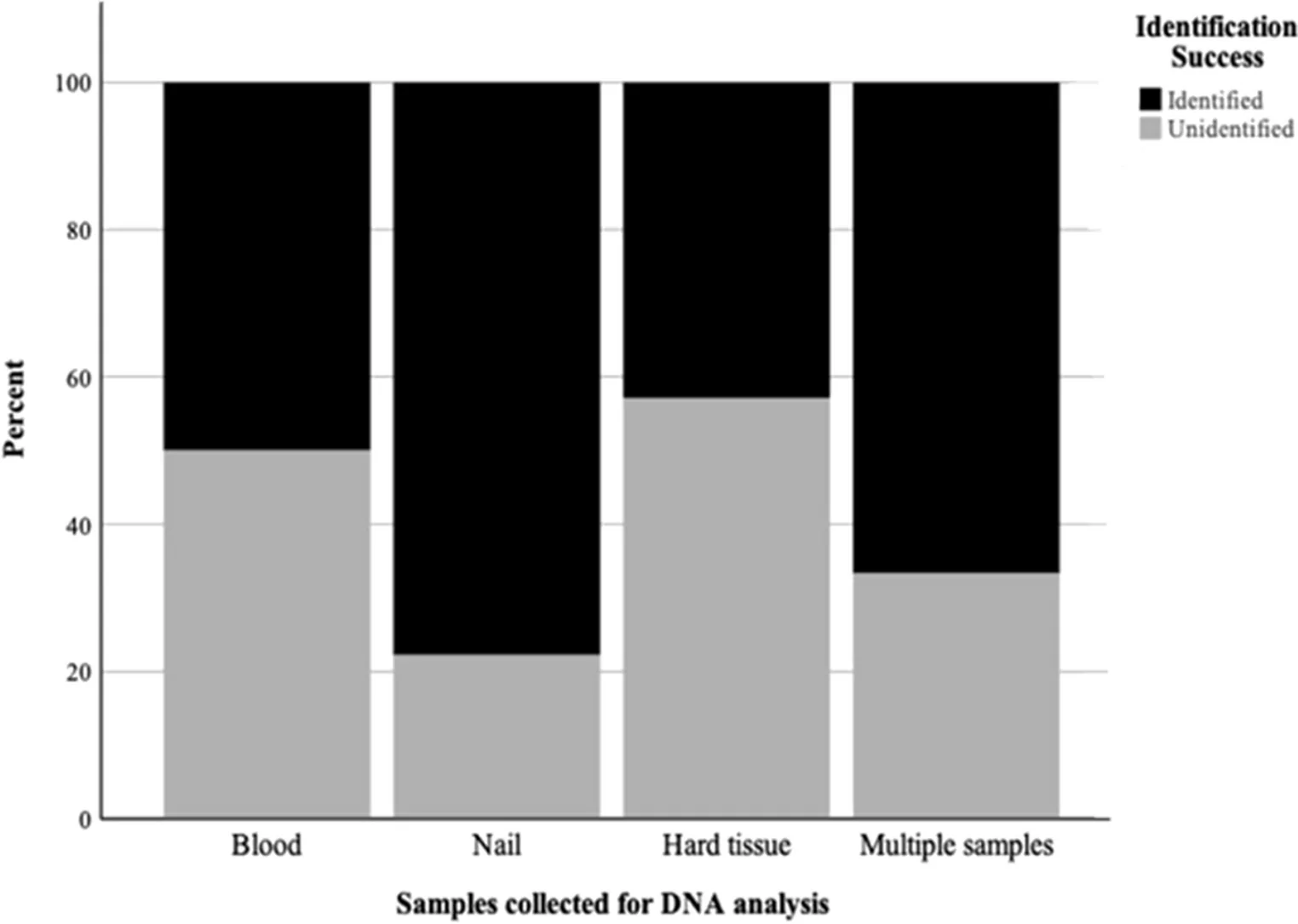
Success rate per sample type collected for DNA analysis

As detailed in the included case reports, identification was primarily confirmed through visual identification (76.1%; 220/263), followed by DNA profiling (9.3%; 27/263), and fingerprinting (5.2%; 15/263) (Fig. 5). DNA profiling samples were collected in 28.4% of cases (82/289), with blood being the most common tissue collected (59.8%; 49/82), followed by nails (12.2%; 10/82) and hard tissue (8.5%; 7/82) samples. Of the hard tissue samples collected during the post-examination, these were reported to include two teeth and five bones. Multiple sample types were collected in the remaining 16 cases. Despite the number of samples collected, successful identification using DNA analysis was only achieved in 32.9% (27/82) of the cases and reported as such in the included case reports. Based on the reported DNA results, it was noted that nails performed the best for identification success rate per sample type, followed by cases where multiple sample types were collected, for example blood collected alongside hard tissue samples (Fig. 6).

## Discussion

The burden of unidentified decedents from aquatic environments in South Africa is poorly reported, as is the situation with many countries (24). This study is the first to retrospectively assess caseloads for drowning in South Africa by using Salt River Mortuary’s post-mortem database and associated case reports. The aim was to evaluate the reported case information to investigate PMSI, body recovery, and identification methods alongside their associated success.

From 2017 to 2021, 289 aquatic-related deaths (1.5% of the mortuary’s caseload) were admitted into Salt River Mortuary for post-mortem examinations. Compared to other countries, this represented a lower percentage of overall mortuary caseload [28, 29]. This finding is likely correlated to the high incidence of other unnatural deaths in the region [30]. Cape Town has one of the highest murder rates globally [31] and a significant number of road traffic accident fatalities [32, 33]. Consequently, the proportion of drownings and other aquatic-related deaths may be disproportionately low when compared to the overall number of unnatural deaths that were admitted for post-mortem examination. However, when comparing the number of drownings per capita for the region, this was found to be not much lower compared to other low- to middle- income countries [34].

Of the included cases, most decedents were reported to be recovered from the ocean. In addition, PMSI was longer in cases recovered from the ocean and the post-mortem identification success rates were also lowest for these cases (87.2%) compared to natural freshwater (90.5%) and urban water sources (98.6%) environments (Fig. 4). Along with the information regarding the reported circumstances of these deaths, each of the above-mentioned findings are discussed in detail below.

### Body recovery: location

From included cases (*N* = 289), it was reported that 48.4% of decedents were recovered from the ocean, whilst 26.0% were from freshwater, and 25.6% from urban water sources (Fig. 3).

This distribution contrasts with findings from North America, where 82.0% of cases between 2008 and 2020 were related to freshwater sources and only 18.0% from saltwater [35]. Similarly, a large-scale study on unintentional drowning mortality rates across 60 countries reported that most decedents were recovered from freshwater [36]. This can be a factor of coastline coverage, which is important to note since the two cited studies failed to specify the inclusion of coastal towns and cities, thus, potentially introducing bias towards major inland cities. Furthermore, a systematic literature review on coastal drownings in Australia by Koon et al. (2021) states that in the 57 multi-water type studies included in their review, coastal drownings accounted for between 10 and 40% of drowning cases reported [37]. Although this percentage is still lower than what was observed in the current study, the upper range is more closely aligned. As raised earlier, these discrepancies with other countries may also relate to differences in overall burden of unnatural deaths, which can be exacerbated by infrastructural, procedural and legal differences.

### Body recovery: PMSI

PMSI for the included cases were documented in the reports as using ‘last seen alive’ information, and where this was not available, a PMSI estimation may have been done through forensic anthropological examination, which would have been requested on the discretion of the forensic pathologist. If neither of the aforementioned were documented, then PMSI was noted as ‘undetermined’. For the analysed case reports, it was found that decedents recovered from the ocean were documented to have a significantly longer PMSI compared to those from natural freshwater sources (Table 3). In freshwater cases, all with a known PMSI (noted as such in the report) were exposed to the environment for less than 15 days. One explanation for the longer PMSI associated with ocean cases may be due to the challenge of recovering decedents from this environment. Recovering decedents from the ocean presents distinct challenges due to its unique taphonomic conditions, vastness, and unpredictable current movements [27, 38]. Strong currents and tides can cause bodies to drift far from their original location, and for body elements or regions to dislodge in the form of natural dismemberment with scavengers also being a consideration [39].

Considering body completeness further, a correlation between reported PMSI and the state of decomposition (also documented) were investigated. A shorter PMSI was significantly associated with more body completeness (*p* < 0.001) and less degradation (*p* < 0.001). Notably, all partial and skeletonised individuals either had a reported PMSI greater that 15 days, or PMSI was noted as undetermined. This finding aligns with other aquatic decomposition studies where PMSI has been found to greatly impact body completeness and the decomposition state [21, 40–42].

Decomposition research in aquatic ecosystems are frequently avoided due to the complex nature of performing the experiments and the logistical barriers they pose [42–47]. Additionally, the few available published, prospective studies used non-human animal analogues to investigate decomposition patterns, which can be difficult to extrapolate to human cases [27, 48–50]. Contrasting results have also been noted with some studies indicating that decomposition occurs slower in aquatic environments compared to terrestrial environments [21, 51, 52]. Of note is that the correlation between PMSI and decomposition appears less clearly delineated in ocean cases than compared to freshwater ones [23]. This may be influenced by factors like depth, displacement of the body, scavenging, and experimental design for prospective oceanic studies [53]. This does highlight that estimating PMSI from the state of the body alone through common taphonomic methods is extremely challenging, and should be done cautiously.

In this retrospective case review study, it must be acknowledged that PMSI was documented from what was shared within the case reports by forensic pathologists who conducted the post-mortem examination of decedents. In these case reports, PMSI was as either well-known from ‘last seen alive’ information or was documented as an estimate based on ancillary investigations (e.g. forensic anthropological analysis). Therefore, PMSI as noted from the case reports included in this study may not be an accurate reflection of the true PMSI. Regardless, the retrospective review clearly showcased that the discovery and recovery of decedents from the ocean was not done as timeously as compared to natural fresh water or urban sources.

### Decedent identification methods

The most common forms of decedent identification as reported were visual identification followed by fingerprinting and DNA profiling (Fig. 5, results). These findings correlate well with what is common in South Africa with visual identification being preferred, fingerprinting used occasionally, and DNA analysis rarely attempted. The limited use of DNA is due to lack of resources, degraded samples, and absence of reference samples for identification. Specifically, comparative antemortem information necessary to confirm an identity is often difficult to obtain given the large number of undocumented non-South African citizens residing in South Africa and the lack of database for identifying missing persons [54]. Therefore, there is a high number of decedents that enter South Africa’s mortuary that remain unidentified.

For aquatic-related deaths in Cape Town, it was found that 9.0% of decedents that entered Salt River Mortuary remained unidentified (Table 2). This percentage is similar to the mortuary’s general unidentified rate (9.2%) and the Pretoria medico-legal laboratory (9.0%) [55].

When considering aquatic-related deaths, several factors may contribute to lower identification rates for these cases. Water is not ideal for preservation and there can be loss of identifiable features, such as facial characteristics, unique body markings, and fingerprints that are often used for identification [21, 41]. In addition, environmental factors such as temperature, salinity, currents, and scavenging can accelerate the decomposition process, thus, further complicating identification efforts [44, 56]. Upon reviewing the data on reported identification methods and associated environmental and post-mortem information, it was found that there was a significant association between decedent recovery location and both the alleged identity (*p* < 0.001) and confirmed final identification (*p* = 0.02) depending if a decedent was recovered from an urban, freshwater, or ocean environment (Table 2). More cases from the ocean were recovered without an alleged identity (*n* = 63) compared to urban water sources (*n* = 29). Cases from the ocean were also less likely to have their identities confirmed following a post-mortem examination and death investigation. For decedents recovered from the ocean, the identification rate was found to be 85.0%, compared to 98.6% for urban water sources and 88.0% for natural freshwater (Fig. 4).

Longer PMSIs were correlated with ocean environments and advanced decomposition, which both likely contributed to identification delays. In cases of advanced decomposition, visual identification cannot be used given the significant tissue loss that occurs. In addition, and due to the tissue loss, DNA preservation is negatively affected, thus impacting successful DNA profiling [47, 57–61]. Furthermore, as raised previously, literature on the identification of decedents recovered from various aquatic environments is limited [21, 40–42]. As the PMSI in this study was restricted by limited data availability (i.e. restricted to what was reported), it was not possible to determine the value of specific identification methods for shorter and longer PMSIs. However, as with terrestrial cases, longer post-mortem intervals often require more specialised methods (anthropology, odontology and molecular) to aid identification.

Samples for DNA profiling were collected in 82 of the 289 cases and were reported as successful in 27 cases. For these, hard tissues samples (bone, teeth, nails) showed potential for DNA recovery, but sample collection was inconsistent. As per the frequency of DNA sample collection, limited resources and capacity often prevent forensic DNA profiling, and when attempted, success rates are low due to the frequent absence of reference samples. In parallel, DNA preservation may be sub-optimal, and depends on intrinsic and extrinsic factors affecting anatomical structures [62, 63]. Therefore, suitability of sample collection choice is influenced by body condition and recovery environment. Blood and soft tissue are frequently degraded, or absent in aquatic or advanced PMSI cases [64]. While hard tissues and nails can yield viable DNA profiles for aquatic-related deaths, the results of the retrospective case review do not allow definitive conclusions on the most effective sample type for different submerged environments—though external guidance is available [65].

### Circumstances surrounding death

Most decedents were reported to be male (74.1%) and aged 18–35, which is similar to general admission rates at Salt River Mortuary (75.1%) [30, 54] and international literature. The median age of 18–35 years for decedents were also similar to generally admissions at Salt River Mortuary [23]. However, compared to other South African mortuaries an increase in decedents under three years of age (6.89% to 10.4%) and over 60 years of age (5.3% to 10.7%) was observed for included aquatic-related cases [30], suggesting increased risk for these ages.

Age as a risk factor for drowning is well documented. In South Africa studies revealed high proportions of drownings in children under twelve in rural areas and under 18 in urban areas [66, 67]. In the Netherlands, the highest incidence of accidental drownings was for individuals under nine years of age and those aged between 50 and 59 years old [68]. Further to this, an American study showed that most individuals recovered from urban water sources were also reported to be under the age of three [54]. In contrast, no distinction was made for adults within the median age range, where drownings are reported for both natural freshwater and the ocean [69].

As per manner of death, Lu et al. (2010) found that globally, 58.0% of aquatic-related deaths were accidental, 24.5% were suicide, and 16.6% were undetermined [70]. In the retrospective review of Salt River Mortuary cases, this study found that half of the deaths were reported as accidental (51.9%), with 27.3% remaining undetermined or still under investigation. Although accidental deaths were seemingly high, a larger number of cases where identity was confirmed had an undetermined cause or manner of death. This finding is likely attributed to the fact that investigations were still ongoing at the time of the study.

Homicide as a manner of death was reported for 10.4% of cases, whereas 6.6% were documented as natural, and 3.8% were presumed suicide. The higher homicide rate correlates with high crime rate and murder in South Africa, particularly in Cape Town [31]. This is further highlighted when comparing to other published studies where accidental is reported more frequently as a manner of death. For example, a study in Germany documents that 72.1% of aquatic-related deaths were accidental, 18.2% were suicide, 2.4% were homicides, and only 7.3% remained undetermined [30]. In addition, statistics from Finland report that 94.3% of decedents recovered from aquatic environments were diagnosed as accidental drowning, with 3.1% due to natural causes and 2.6% from other injury-related causes [71].

The figures from the northern hemisphere countries indicate the large difference observed between developed and developing countries regarding the manner and cause of death and how often a diagnosis of death is provided. The disparity in diagnostic outcomes highlights the impact that varying levels of forensic expertise, resource availability, and investigative rigor contribute to case resolution. In countries like Germany and Finland, most aquatic-related deaths are clearly categorised, with high percentages of accidental deaths and relatively low percentages of undetermined cases. Conversely in regions such as South Africa, a considerable portion of aquatic-related medico-legal deaths are undetermined, reflecting the challenges faced by forensic pathology services and the potential influence of high crime rates in South Africa. Further to this, the high number of accidental deaths highlight the continuing need for safe-swimming programmes, especially in developing countries where formal education and resources are lacking.

### Study limitations

These data were obtained directly from case files without any ability to verify or standardise the collected information regarding cause-of-death, manner-of-death, PMSI, and the identification outcome. There was a lack of organised documentation available from the State forensic authorities on the recovery location, the identification methods and processes for decedents recovered from aquatic environments. Due to the reliance of data recorded from the case files, certain information and more detailed analyses were not possible. For example, it would have been interesting and informative to analyse these data with other variables such as temperature, seasonality, socioeconomic factors and/or activities during the time of death (e.g. maritime work, recreational beach activities, tourism). These gaps were identified and may be pursued in the future using a prospective approach. This would add to the translatability of these findings towards tailored prevention strategies. The aforementioned combined represented a hindrance in ensuring comprehensive and consistent data collection. Additionally, many cases lacked recovery location data, and numerous variables were missing even when the location was documented, which led to divergent results and reporting inconsistency.

The lack of consistent reporting raises an important need for more standardised protocols for documenting aquatic-related deaths. However, as a retrospective study, missing data are an inherent limitation of this type of analysis. However, in the current study, no significant differences were observed in the proportion of cases with missing data (of each assessed categorical variable) for each water source assessed. As such the data were assumed to be missing completely at random and cases with missing data for specific variables were excluded from analysis on that variable. This approach, although suffering from limited sample sizes, should alleviate some study bias.

For urban water sources it is acknowledged that the biological and chemical conditions of sewage and swimming pools are different, therefore, the results from this category would require further interrogation in the future regarding their impact on degradation of tissue and DNA. Furthermore, in the absence of prospective testing of these water sources, it is not possible to draw firm conclusions about potential differences in urban water supplies, as such testing fell outside the scope of this study.

## Conclusions

Aquatic-related deaths are rising globally, driven in part by increased migration through water routes. Salt River Mortuary, like many medico-legal facilities in Africa, manages high caseloads and a higher proportion of unidentified decedents. Unidentified cases carry heavy demands on mortuaries, and in the case of Cape Town—a coastal city—aquatic-related deaths form part of this demand. To date, no prior research has examined aquatic-related deaths in South Africa. Therefore, the aim was to review the mortality data from Salt River Mortuary to identify relevant cases linked to aquatic deaths.

The findings of the retrospective case revealed several important factors in the investigation of aquatic-related deaths. Results revealed significantly lower identification rates for ocean recoveries, likely linked to longer PMSIs, and confirmed strong correlations between PMSI, body completeness, and decomposition. These findings highlight the need for specialised identification methods and demonstrate the value of retrospective medico-legal analyses in shaping forensic protocols. The absence of standard procedures for handling waterlogged remains leaves identification and sampling decisions to individual pathologists, creating inconsistency. Ongoing research and procedural development are essential to improve post-mortem identification, ensure more accurate case resolutions, and better understand the circumstances surrounding aquatic deaths. These data illustrate the value drawn from retrospective medico-legal case work to improve forensic science approaches and identify forensically relevant research questions.

## Key Points

1. Oceanic cases had lower identification rates (87.2%) compared to freshwater (90.5%) and urban sources (98.6%), likely due to longer post-mortem submersion intervals (PMSI) and advanced decomposition.
2. Visual identification dominates in South Africa, with DNA profiling used infrequently due to resource constraints and difficulty collecting antemortem reference samples.
3. Oceanic recovery faces challenges like strong currents, leading to longer PMSIs and more decomposition, complicating identification efforts.
4. Specialised methods like forensic anthropology and DNA analysis are crucial for cases with longer PMSIs or from complex aquatic environments like the ocean.

## Electronic Supplementary Material

Below is the link to the electronic supplementary material.

Supplementary Material 1 (DOCX 14.7 KB)

## References

[1] Boss PG. Ambiguous loss: working with families of the missing. Fam Process. 2002;41:14–7. 10.1111/J.1545-5300.2002.40102000014.X. 11924081

[2] Suwalowska H, Ali J, Rangel de Almeida J, Fonseca SA, Heathfield LJ, Keyes CA, et al. The Nobodies: unidentified dead bodies—a global health crisis requiring urgent attention. Lancet Glob Health. 2023;11:e1691–3. 10.1016/S2214-109X(23)00420-5. 37774720

[3] Cattaneo C, Tidball Binz M, Penados L, Prieto J, Finegan O, Grandi M. The forgotten tragedy of unidentified dead in the Mediterranean. Forensic Sci Int. 2015;250:e1-2. 10.1016/J.FORSCIINT.2015.02.007. 25770023

[4] Pekka S, Knight B. Knight’s Forensic Pathology. Fourth. 2016.

[5] World Drowning Prevention Day | United Nations. n.d. https://www.un.org/en/observances/drowning-prevention-day (accessed June 6, 2024).

[6] Scarr JP, Buse K, Norton R, Meddings DR, Jagnoor J. Tracing the emergence of drowning prevention on the global health and development agenda: a policy analysis. Lancet Glob Health. 2022;10:e1058-66. 10.1016/S2214-109X(22)00074-2. PMC9197772 35461520

[7] Words into Action Guidelines. National Disaster Risk Assessment Hazard 2. Tsunami Hazard and Risk Assessment n.d.

[8] International Organization for Migration n. d. https://missingmigrants.iom.int/ (accessed November 22, 2022).

[9] Garcia Borja A, Abdelazim M. The Situation of Migrant Deaths in the Mediterranean in 2021. Geneva: 2021.

[10] Watherston J, Watson J, Bruce D, Ward J, McNevin D. Efficient DNA profiling protocols for disaster victim identification. Forensic Sci. 2021;1:148–70. 10.3390/FORENSICSCI1030014.

[11] Fortuin J, Karangwa I, Mahlalela N, Robertson C. A South African epidemiological study of fatal drownings. Int J Environ Res Public Health. 2022;19:2016–21. 10.3390/ijerph192215121. PMC9690020 36429836

[12] Annual report downloads | NSRI n.d. https://www.nsri.org.za/who-we-are/annual-report-downloads-1 (accessed November 30, 2022).

[13] Beachgoers retrieve body of elderly woman at Ramsgate beach on KZN South Coast. n.d. https://www.iol.co.za/mercury/news/beachgoers-retrieve-body-of-elderly-woman-at-ramsgate-beach-on-kzn-south-coast-68fe412f-e8b0-4d34-b2d4-0bbd92828880 (accessed November 30, 2022).

[14] Bodies of a mother. and daughter recovered after a double drowning on the KZN South Coast n.d. https://www.iol.co.za/dailynews/news/kwazulu-natal/bodies-of-a-mother-and-daughter-recovered-after-a-double-drowning-on-the-kzn-south-coast-104a9ad0-bdd6-405f-b7dd-b7d5b9137d53 (accessed November 30, 2022).

[15] Body of man who went missing last weekend found at Sunrise Beach. n.d. https://www.iol.co.za/capeargus/news/body-of-man-who-went-missing-last-weekend-found-at-sunrise-beach-ac80eff1-d711-4f54-8a53-add080c4ed33 (accessed November 30, 2022).

[16] Body savaged by sharks found on beach in South Africa | News. | The Times n.d. https://www.thetimes.co.uk/article/body-savaged-by-sharks-found-on-beach-in-south-africa-kvnmz7zx7 (accessed November 30, 2022).

[17] Bodies of two men found on the rocks at Sea Point. beach n.d. https://www.iol.co.za/capeargus/news/bodies-of-two-men-found-on-the-rocks-at-sea-point-beach-a3f2b2df-a622-49bb-87fa-478928c10f90 (accessed November 30, 2022).

[18] Bodies of man and woman with gunshot wounds found on Strandfontein. beach n.d. https://www.iol.co.za/capetimes/news/bodies-of-man-and-woman-with-gunshot-wounds-found-on-strandfontein-beach-5796c8bb-ea58-4a70-b56b-8b93d91b52e0 (accessed November 30, 2022).

[19] Gangsters turn Cape. Town beauty spot into corpse dumping ground | World | The Times n.d. https://www.thetimes.co.uk/article/gangsters-turn-cape-town-beauty-spot-into-corpse-dumping-ground-kzz93h395 (accessed November 30, 2022).

[20] Migration Data in the Southern African Development Community (SADC). | Migration data portal n.d. https://www.migrationdataportal.org/regional-data-overview/southern-africa (accessed December 1, 2022).

[21] Ambade VN, Kukde HG, Malani A, Tumram NK, Borkar JL, Batra AK, et al. Decomposed and non-decomposed bodies retrieved from water: a comparative approach. Med Sci Law. 2013;53:12–8. 10.1258/msl.2012.012037. 23155119

[22] Palmer C. Estimating the impact of laminar flow on the pattern and rate of decomposition in aquatic environments—is there a better way of modeling decomposition? J Forensic Sci. 2020;65:1601–9. 10.1111/1556-4029.14441. 32347978

[23] van Daalen MA, de Kat DS, Oude Grotebevelsborg BFL, de Leeuwe R, Warnaar J, Oostra RJ, et al. An aquatic decomposition scoring method to potentially predict the postmortem submersion interval of bodies recovered from the North Sea. J Forensic Sci. 2017;62:369–73. 10.1111/1556-4029.13258. 28247448

[24] Martlin BA, Anderson GS, Bell LS. A review of human decomposition in marine environments. Can Soc Forensic Sci J. 2023;56:92–121. 10.1080/00085030.2022.2135741.

[25] Butler JM, Willis S. Interpol review of forensic biology and forensic DNA typing 2016–2019. Forensic Sci Int. 2020;2:352–67. 10.1016/J.FSISYN.2019.12.002. PMC7770417 33385135

[26] Budowle B, Bieber FR, Eisenberg AJ. Forensic aspects of mass disasters: strategic considerations for DNA-based human identification. Leg Med. 2005;7:230–43. 10.1016/J.LEGALMED.2005.01.001. 15975517

[27] Finaughty C, Gibbon VE, Speed B, Heathfield LJ. Lost at sea: a pilot study investigating DNA recovery from teeth in a South African natural marine environment. Forensic Sci Int Genet Suppl Ser. 2019;7:580–1. 10.1016/J.FSIGSS.2019.10.097.

[28] Prameprart M, Lim A, Tongkumchum P. Modeling unintentional drowning mortality rates in Thailand, 2000–2009. Asia Pac J Public Health. 2015;27:NP2471-9. 10.1177/1010539513488796/ASSET/IMAGES/LARGE/10.1177_1010539513488796-FIG4.JPEG. 23761591

[29] Shotar AM, Halalsheh M, Shatnawi R, Abu-El-rub H, Hussein NA, Shoter S, et al. Epidemiological analysis of drowning deaths among different groups in Jordan - a retrospective study (2015–2019). Med Arch. 2022;76:49. 10.5455/MEDARH.2022.76.49-54. PMC8978540 35422567

[30] Schneppe S, Dokter M, Bockholdt B. Macromorphological findings in cases of death in water: a critical view on “drowning signs.” Int J Legal Med. 2020. 10.1007/s00414-020-02469-9. PMC7782435 33237456

[31] Western Cape Government on murder statistics | South African Government n. d. https://www.gov.za/news/media-statements/western-cape-government-murder-statistics-17-nov-2023 (accessed January 12, 2024).

[32] Albertyn Z, Mathews S, Coetzee D, Bastiaan Van As A. Morbidity and mortality from road traffic crashes in children in Metro West, City of Cape Town – 2014. Int J Inj Contr Saf Promot. 2019;26:115–23. 10.1080/17457300.2018.1508165. 30239263

[33] Toit C, Du. Cape Town road traffic accident analysis: Utilising supervised learning techniques and discussing their effectiveness 2022.

[34] Fortuin J, Karangwa I, Mahlalela N, Robertson C. A south African epidemiological study of fatal drownings: 2016–2021. Int J Environ Res Public Health. 2022;19:2016–21. 10.3390/IJERPH192215121. PMC9690020 36429836

[35] Girela-López E, Beltran-Aroca CM, Dye A, Gill JR. Epidemiology and autopsy findings of 500 drowning deaths. Forensic Sci Int. 2022. 10.1016/J.FORSCIINT.2021.111137. 34894613

[36] Lin C-Y, Wang Y-F, Lu T-H, Kawach I. Unintentional drowning mortality, by age and body of water: an analysis of 60 countries. Inj Prev. 2015. 10.1136/injuryprev-2013-041110. PMC4501178 24944343

[37] Koon WA, Peden AE, Lawes JC, Brander RW. Mortality trends and the impact of exposure on Australian coastal drowning deaths, 2004–2021. Aust N Z J Public Health. 2023;47:100034. 10.1016/J.ANZJPH.2023.100034. 36963121

[38] Martlin BA, Bell LS. Water-related fatalities: an examination of body displacement and recovery patterns in British Columbia, Canada. J Forensic Sci. 2023;00:1–20. 10.1111/1556-4029.15395. 37789521

[39] Ruffell A, Pringle JK, Cassella JP, Morgan RM, Ferguson M, Heaton VG, et al. The use of geoscience methods for aquatic forensic searches. Earth-Sci Rev. 2017;171:323–37. 10.1016/J.EARSCIREV.2017.04.012.

[40] Palazzo C, Pelletti G, Fais P, Giorgetti A, Boscolo-Berto R, Gaudio RM, et al. Application of aquatic decomposition scores for the determination of the post mortem submersion interval on human bodies recovered from the Northern Adriatic Sea. Forensic Sci Int. 2021;318:110599. 10.1016/J.FORSCIINT.2020.110599. 33279762

[41] Caruso JL. Decomposition changes in bodies recovered from water. Acad Forensic Pathol. 2016;6:19. 10.23907/2016.003. PMC6474513 31239870

[42] Franceschetti L, Palamenghi A, Mazzarelli D, Cappella A, Gibelli DM, De Angelis D, et al. Taphonomic study on drowned victims in a non-sequestered aquatic environment in the Mediterranean Sea. Int J Legal Med. 2022;136:887–95. 10.1007/S00414-021-02745-2/TABLES/5. 34802063

[43] Leggio A, Tarzia P, Introna F. The role of forensic anthropology in the medico-legal investigation of remains recovered at sea: analysis of a case. Forensic Sci. 2021;1(3):138–47. 10.3390/FORENSICSCI1030013.

[44] Webber J, Moran K, French C, Fozard F, Pearless O. Fatal coastal drowning incidents: a 10-year review of body recovery times in New Zealand. Forensic Sci Int. 2020;317:110573. 10.1016/J.FORSCIINT.2020.110573. 33214011

[45] Pesquero MD, Bell LS, Fernández-Jalvo Y. Skeletal modification by microorganisms and their environments. Hist Biol. 2017;30:882–93. 10.1080/08912963.2017.1371713.

[46] Stock MK, Winburn AP, Burgess GH. Skeletal indicators of shark feeding on human remains: evidence from Florida forensic anthropology cases. J Forensic Sci. 2017;62:1647–54. 10.1111/1556-4029.13470. 28464294

[47] Ellingham STD, Perich P, Tidball-Binz M. The fate of human remains in a maritime context and feasibility for forensic humanitarian action to assist in their recovery and identification. Forensic Sci Int. 2017;279:229–34. 10.1016/j.forsciint.2017.07.039. 28934682

[48] Bartlett AH. The effects of various salinity concentrations in submerged neonatal pigs. University of California; 2020.

[49] Matuszewski S, Konwerski S, Frątczak K, Szafałowicz M. Effect of body mass and clothing on decomposition of pig carcasses. Int J Legal Med. 2014;128:1039–48. 10.1007/S00414-014-0965-5. PMC4196037 24487775

[50] Eriksen AMH, Matthiesen H, Kontopoulos I, Gregory D, Snoeck C, Zhang G, et al. Rapid loss of endogenous DNA in pig bone buried in five different environments. Archaeometry. 2020;62:827–46. 10.1111/ARCM.12553.

[51] Heaton V, Lagden A, Moffatt C, Simmons T. Predicting the postmortem submersion interval for human remains recovered from U.K. waterways. J Forensic Sci. 2010;55:302–7. 10.1111/j.1556-4029.2009.01291.x. 20102465

[52] Fink A. Estimating Postmortem Intervals of Human Remains Recovered in Mid-Western Waterways: A Test of Terrestrial and Aquatic Body Scoring Methods. Masters Theses; 2017.

[53] De Donno A, Campobasso CP, Santoro V, Leonardi S, Tafuri S, Introna F. Bodies in sequestered and non-sequestered aquatic environments: a comparative taphonomic study using decompositional scoring system. Sci Justice. 2014;54:439–46. 10.1016/j.scijus.2014.10.003. 25498931

[54] Reid KM, Martin LJ, Heathfield LJ. Bodies without names: a retrospective review of unidentified decedents at Salt River Mortuary, Cape Town, South Africa, 2010–2017. S Afr Med J. 2020;110:223–8. 10.7196/SAMJ.2020.v110i3.14192. 32657700

[55] Evert L, Rossouw SH. Unidentified bodies in forensic pathology practice in South Africa: Demographic and Medico-Legal Perspectives. University of Pretoria; 2011.

[56] de Boer HH, Blau S, Delabarde T, Hackman L. The role of forensic anthropology in disaster victim identification (DVI): recent developments and future prospects. Forensic Sci Res. 2019;4:303. 10.1080/20961790.2018.1480460. PMC6968550 32002489

[57] Anderson GS, Bell LS. Deep coastal marine taphonomy: investigation into carcass decomposition in the Saanich Inlet, British Columbia using a baited camera. PLoS One. 2014;9:e110710. 10.1371/journal.pone.0110710. PMC4203822 25329759

[58] Cartozzo C, Singh B, Swall J, Simmons T. Postmortem submersion interval (PMSI) estimation from the microbiome of sus scrofa bone in a freshwater lake. J Forensic Sci. 2021;66:1334–47. 10.1111/1556-4029.14692. 33818789

[59] Randall S, Cartozzo C, Simmons T, Swall JL, Singh B. Prediction of minimum postmortem submersion interval (PMSImin) based on eukaryotic community succession on skeletal remains recovered from a lentic environment. Forensic Sci Int. 2021. 10.1016/j.forsciint.2021.110784. 33864992

[60] Helm K, Matzenauer C, Neuhuber F, Monticelli F, Meyer H, Pittner S, et al. Suitability of specific soft tissue swabs for the forensic identification of highly decomposed bodies. Int J Legal Med. 2021;135:1319–27. 10.1007/s00414-021-02601-3. PMC8205910 33880634

[61] Inkret J, Podovšovnik E, Zupanc T, Pajnič IZ. Nails as a primary sample type for molecular genetic identification of highly decomposed human remains. Int J Legal Med. 2020;134:1629–38. 10.1007/s00414-020-02289-x. 32372234

[62] Raffone C, Baeta M, Lambacher N, Granizo-Rodríguez E, Etxeberria F, de Pancorbo MM. Intrinsic and extrinsic factors that may influence DNA preservation in skeletal remains: A review. Forensic Sci Int. 2021;325:110859. 10.1016/J.FORSCIINT.2021.110859. 34098475

[63] Latham KE, Miller JJ. DNA recovery and analysis from skeletal material in modern forensic contexts. Forensic Sci Res. 2019;4:51. 10.1080/20961790.2018.1515594. PMC6427720 30915417

[64] Thakar MK, Joshi B, Shrivastava P, Raina A, Lalwani S. An assessment of preserved DNA in decomposed biological materials by using forensic DNA profiling. Egypt J Forensic Sci. 2019;9:1–9. 10.1186/S41935-019-0151-5/FIGURES/6.

[65] Finaughty C, Heathfield LJ, Kemp V, Márquez-Grant N. Forensic DNA extraction methods for human hard tissue: A systematic literature review and meta-analysis of technologies and sample type. Forensic Sci Int Genet. 2023;63:102818. 10.1016/J.FSIGEN.2022.102818. 36502616

[66] Morris NK, Du Toit-Prinsloo L, Saayman G. Drowning in Pretoria, South Africa: a 10-year review. J Forensic Leg Med. 2016;37:66–70. 10.1016/J.JFLM.2015.10.010. 26599373

[67] Garenne M, Kahn K, Tollman S, Gear J. Causes of death in a rural area of South Africa: an international perspective. J Trop Pediatr. 2000;46:183–90. 10.1093/TROPEJ/46.3.183. 10893926

[68] Bierens J, Hoogenboezem J. Fatal drowning statistics from the Netherlands-an example of an aggregated demographic profile. BMC Public Health. 2021. 10.1186/s12889-022-12620-3. PMC8851711 35177025

[69] Mott TF, Latimer KM. Prevention and treatment of drowning. Am Fam Physician. 2016;93:576–82. 27035042

[70] Lu TH, Lunetta P, Walker S. Quality of cause-of-death reporting using ICD-10 drowning codes: a descriptive study of 69 countries. BMC Med Res Methodol. 2010;10:1–6. 10.1186/1471-2288-10-30/COMMENTS. PMC2858216 20374660

[71] Lunetta P, Penttila A, Sajantila A. Circumstances and macropathologic findings in 1590 consecutive cases of bodies found in water. Am J Forensic Med Pathol. 2002;23:371–6. 10.1097/00000433-200212000-00015 12464816

