# Supplementary material for: A retrospective case review of decedents recovered from aquatic environments in Cape Town, South Africa
Source: Forensic Sci Med Pathol. 2026 May 28;22(3):1037–50. doi: 10.1007/s12024-026-01229-2 (PMC13672400; doi:10.1007/s12024-026-01229-2)
Supplement: Supplementary file 1 [file 12024_2026_1229_MOESM1_ESM.docx]

**Supplementary Table A**

| **Location (coordinates)** |  | | | | | | | | | | | | | |
| --- | --- | --- | --- | --- | --- | --- | --- | --- | --- | --- | --- | --- | --- | --- |
| **Water type** | Indian Ocean | Atlantic Ocean | | | | | Lake | | | | | River | | Swimming pool |
|  | Bath | Other (specify): | | | | | | | | | | | | |
| **Cause of death** |  | | | | | | | | | | | | | |
| **Suspected manner of death** | Homicide | Suicide | | | | | Accidental | | | | | Natural | | Undetermined |
| **PMSI** |  | | | | | | | | | | | | | |
| **Method used to determine PMSI** |  | | | | | | | | | | | | | |
| **Timeline** | Date admitted to SRM: | | | | | Date of autopsy: | | | | | Date released from mortuary: | | | |
|  | Date of death declaration: | | | | | | | | Total days past: | | | | | |
| **Body condition** | Full | Partial (Specify): | | | | | | | | | | | | |
|  | Fresh | | | | Decomposed | | | | | | | | Skeletonised | |
|  | Describe: | | | | | | | | | | | | | |
| **Samples collected for identification** | Blood | | Teeth | | | | | Bone | | | | Nail | | |
|  | Other (Specify) | | | | | | | | | | | | | |
| **Identified** | Yes | | | | | | | | | No | | | | |
|  | Before autopsy | | | After autopsy | | | | | |  |  |  |  |  |
| **If identified, how?** |  | | | | | | | | | | | | | |
| **Date of birth** |  | | | | | | | | | | | | | |
| **Sex** | Male | | | | | | | | Female | | | | Unknown | |
